# Modeling the Research Landscapes of Artificial Intelligence Applications in Diabetes (GAP_RESEARCH_)

**DOI:** 10.3390/ijerph17061982

**Published:** 2020-03-17

**Authors:** Giang Thu Vu, Bach Xuan Tran, Roger S. McIntyre, Hai Quang Pham, Hai Thanh Phan, Giang Hai Ha, Kenneth K. Gwee, Carl A. Latkin, Roger C.M. Ho, Cyrus S.H. Ho

**Affiliations:** 1 Center of Excellence in Evidence-based Medicine, Nguyen Tat Thanh University, Ho Chi Minh City 700000, Vietnam; giang.coentt@gmail.com; 2 Institute for Preventive Medicine and Public Health, Hanoi Medical University, Hanoi 100000, Vietnam; bach.ipmph@gmail.com; 3 Bloomberg School of Public Health, Johns Hopkins University, Baltimore, MD 21205, USA; carl.latkin@jhu.edu; 4 Institute of Medical Science, University of Toronto, Toronto, ON M5S 1A8, Canada; roger.mcintyre@uhn.ca; 5 Mood Disorders Psychopharmacology Unit, University Health Network, Toronto, ON M5G 2C4, Canada; 6 Department of Psychiatry, University of Toronto, Toronto, ON M5T 1R8, Canada; 7 Department of Toxicology and Pharmacology, University of Toronto, Toronto, ON M5S 1A8, Canada; 8 Institute for Global Health Innovations, Duy Tan University, Da Nang 550000, Vietnam; qhai.ighi@gmail.com (H.Q.P.); haipt.ighi@gmail.com (H.T.P.); 9 Department of Psychological Medicine, Yong Loo Lin School of Medicine, National University of Singapore, Singapore 119228, Singapore; e0012499@u.nus.edu (K.K.G.);; 10 Center of Excellence in Behavioral Medicine, Nguyen Tat Thanh University, Ho Chi Minh City 700000, Vietnam; 11 Institute for Health Innovation and Technology (iHealthtech), National University of Singapore, Singapore 117599, Singapore; 12 Department of Psychological Medicine, National University Hospital, Singapore 119074, Singapore; cyrushosh@gmail.com

**Keywords:** artificial intelligence, machine learning, diabetes, bibliometric, LDA

## Abstract

The rising prevalence and global burden of diabetes fortify the need for more comprehensive and effective management to prevent, monitor, and treat diabetes and its complications. Applying artificial intelligence in complimenting the diagnosis, management, and prediction of the diabetes trajectory has been increasingly common over the years. This study aims to illustrate an inclusive landscape of application of artificial intelligence in diabetes through a bibliographic analysis and offers future direction for research. Bibliometrics analysis was combined with exploratory factor analysis and latent Dirichlet allocation to uncover emergent research domains and topics related to artificial intelligence and diabetes. Data were extracted from the Web of Science Core Collection database. The results showed a rising trend in the number of papers and citations concerning AI applications in diabetes, especially since 2010. The nucleus driving the research and development of AI in diabetes is centered around developed countries, mainly consisting of the United States, which contributed 44.1% of the publications. Our analyses uncovered the top five emerging research domains to be: (i) use of artificial intelligence in diagnosis of diabetes, (ii) risk assessment of diabetes and its complications, (iii) role of artificial intelligence in novel treatments and monitoring in diabetes, (iv) application of telehealth and wearable technology in the daily management of diabetes, and (v) robotic surgical outcomes with diabetes as a comorbid. Despite the benefits of artificial intelligence, challenges with system accuracy, validity, and confidentiality breach will need to be tackled before being widely applied for patients’ benefits.

## 1. Introduction

Diabetes is a chronic medical disease that is characterized by increased levels of blood glucose, which causes microvascular and macrovascular complications with time. This chronic condition is concerning because the prevalence of diabetes has been steadily increasing for the past three decades. In 2014, the worldwide prevalence of diabetes was 8.5% among those aged 18 years and older, a escalation from 4.7% in 1980 [1]. In 2016, an estimated 1.6 million deaths were directly attributable to diabetes alone with World Health Organization (WHO) estimating that diabetes was the seventh leading cause of death in 2016. This figure is expected to rise even further in the future [1].

The global health expenditure on diabetes among people of ages 20 and 79 is expected to rise from USD 376 billion in 2010 to an estimated USD 490 billion in 2030 [2]. All these alarming statistics justify the need for ongoing research and active management to prevent and treat diabetes and its complications to optimize quality of life as well as to reduce the economic healthcare burden.

One of the promising research areas that are ongoing in the field of diabetes, is the use of artificial intelligence (AI). Artificial intelligence is a domain in computer science which accentuates the creation and use of intelligent machines that are able to function and respond like humans. According to a recent 2019 bibliometric study, there has been a tripled fold increase in the number of studies on the applications of AI in the past three years alone, with Diabetes being among the top ten areas of interest [3].

The techniques applied in the research of AI and diabetes include machine learning, artificial neural networks, and natural language processing. These techniques have allowed several applications of AI into the diagnosis and management of diabetes such as the diagnosis of microalbuminuria in type II diabetes patients without the need to measure urinary albumin levels. Microalbuminuria (MA) is a known complication of diabetes and is one of the measures of the renal function in diabetic patients The gold standard of such a diagnosis is to collect 24-hour urine albumin excretion, but with artificial intelligence on the rise, the detection of MA can be done with clinical parameters usually monitored in type II diabetes patients such as age, duration of diabetes, body mass index, and HbA1c (which is the average of blood glucose over the past three months, commonly used to assess diabetic control) [4].

Another promising application of artificial intelligence in diabetes is the development of a model that helps to generate a risk score with the ability to predict future glycemic control in individuals with type II diabetes. Machine learning models have been established and used to forecast the trajectories using clinical parameters such as body mass index, glycated hemoglobin, and triglycerides. This developed model can be used to estimate the patient’s journey with diabetes and determine the follow-up period based on their risk score. Patients with higher risk scores should be followed up more closely compared to those who have lower risk scores to optimize their health outcomes, particularly in diabetes [5].

The evidently increasing interest of academics and practitioners in applications of AI in diabetes management sparked a need for having a comprehensive and up-to-date picture of what has been done in terms of research on this topic, highlighting areas that have been investigated, the emerging research domains, and potential research gaps that need further investigation. Informed readers may, in turn, be able to decide on a better direction for their future researches on the topic. To the best of our knowledge, there has not been a publication looking into AI application in diabetes on a comprehensive, global scale. This study is conducted to fill such gap in literature by adopting a combination of bibliometric approach and more complex analysis of title and abstracts of publications.

## 2. Materials and Methods

### 2.1. Search Strategy

We did a search on the Web of Science (WOS) Core Collection, an online database covering the bibliographic data of various research areas since 1900 [6], and retrieved all papers related to artificial intelligence in diabetes [7]. The full search strategy has been presented in another paper [3]. In this analysis, we selected and retrieved the data on AIs that were related to diabetes in two steps:

(a) Step 1: the publications related to AI in medicine and healthcare were extracted [3];
(b) Step 2: among the papers in step 1, we used terms related to diabetes for identifying studies related to diabetes in AI in health and medicine.

### 2.2. Data Extraction

All data in .txt format were downloaded from the WOS including title, authors’ names, journal, year of publication, affiliations, a total of citation, keywords, and abstracts. All of these data were converted to xls. file (Microsoft Excel) for checking data error. After this, we filtered all downloaded data by sieving out papers that were: (1) not original articles and reviews, (2) unrelated to diabetes and AIs, and (3) not in English. Two researchers worked independently to guarantee the quality of data download and extraction. Any conflict in terms of paper selection was resolved by discussion. The collective dataset was eventually transferred into Stata 14.0 to be further analyzed (STATACorp., College Station, Texas, USA).

### 2.3. Data Analysis

We analyzed the dataset based on the following information: general characteristic (number of papers per year, citations, the total and average number of download publications), keywords (most common keywords and co-occurrence keywords), and text mining (abstract). After we have downloaded and extracted the data, descriptive statistical analysis using Stata was applied to calculate the number of papers by countries mentioned in abstracts. A network graph that illustrated the connection among authors’ keyword co-occurrence network was created by the VOSviewer (version 1.6.8, Center for Science and Technology, Leiden University, Leiden, the Netherlands). For analyzing the contents of the abstracts, exploratory factor analysis (EFA) was employed to identify and visualize the research domains that stem from all content of the abstracts; to highlight research topics or terms most commonly co-occurring with each other, we used the 0.4. Jaccard’s similarity index. Latent Dirichlet Allocation (LDA) was used for classifying papers into corresponding topics [8,9,10,11,12]. Principal component analysis (PCA) was used to create the keyword map as the technique is able to reduce the number of variables, and thus, cluster them into more manageable groups [13]. EFA, LDA, and PCA were conducted using Stata. The analytical techniques for each data type are shown in Table 1.

### 2.4. Ethical Statement

This study used statistics on papers and citations retrieved from the Web of Sciences databases. No human subjects involved, so that this is not subject to ethical review requirements for biomedical research.

## 3. Results

Table 2 gives the basic characteristic of the research papers. There has been an increased interest in studies applying AI to diabetes during 1991–2018. The number of papers has been growing gradually, with two-thirds of the total of 372 papers being published in the 2014–2018 period. Notably, the papers being published in 2009 have the highest total citations, mean cite rate and mean use rate in the last five years (2014–2018).

In terms of study settings (i.e., the country where the study was conducted), there were a total of 36 countries being mentioned in the abstracts of 372 publications (see Table 3). Of those, the United States of America was mentioned 44.1%, followed by Ireland (10.2%) and Italy (6.1%). The top 10 countries accounted for over 80% of the total study settings. Noticeably, in some countries where the prevalence of diabetes is higher than others [14] such as Saudi Arabia (*n* = 3, 1.2%), Egypt (*n* = 1, 0.4%), and United Arab of Emirates (*n* = 2, 0.8%), the number of papers was small compared to others with less prevalence of diabetes like Ireland, Italy, and Japan.

**Figure 1:**
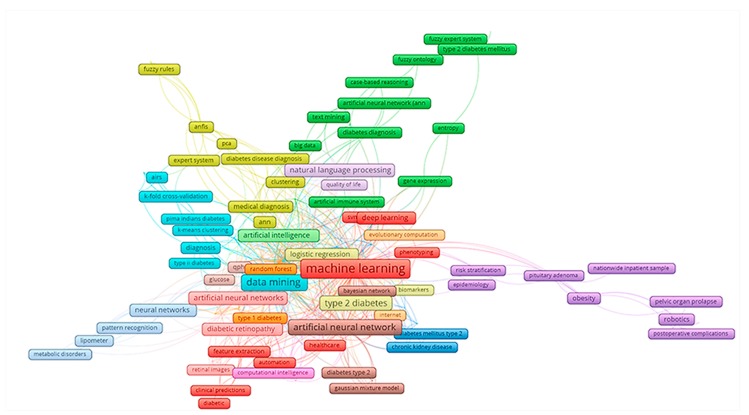
Co-occurrence of most frequent authors’ keywords. The colors of the nodes indicate principal components of the data structure; the size of the node was scaled to the keywords’ occurrences; the thickness of the lines was drawn based on the strength of the association between two keywords.

By analyzing the keywords and abstracts’ contents, it provided us with a clearer comprehension of the scopes of studies and development of research landscapes. Figure 1 describes the co-occurrence of keywords with the most common groups of terms. There were 17 major clusters that emerged from 165 most common keywords with co-occurrence of 5 times and higher. Of which, we could arrange into three major clusters: (1) AI types and its application in diabetes such as red cluster (machine learning, deep learning and clinical predictions), turquoise cluster (data mining and type II diabetes diagnosis), and green cluster (artificial neural network, big data and gene expression in diabetes diagnosis); (2) diabetes types: light yellow cluster and blue cluster (type II diabetes) and orange cluster (type 1 diabetes); (3) robotics in surgery, risk factor (obesity) and epidemiology of diabetes.

As for the content analysis of abstracts, the top 20 emerging research domains that were highlighted via the exploratory factor analysis of abstracts are listed in Table 4**.** Some AI techniques have been most used in this dataset including fuzzy expert system, support vector machine, artificial neural network, and machine learning. Those branches were applied in the following fields of diabetes: (1) clinical prediction (health records collecting or support vector machine modeling for prediction); (2) diabetes management (monitor blood sugar levels); (3) robot-assisted surgery with complication (hypertension, or obesity); (4) the cost of diabetes care.

**Figure 2:**
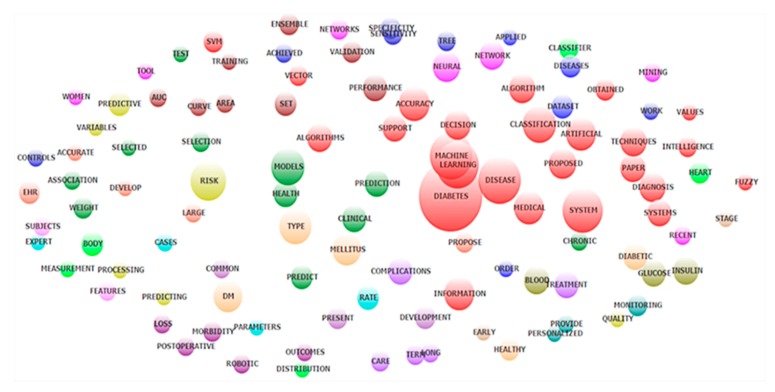
Co-occurrence of most frequent topics emerged from exploratory factor analysis of abstracts contents.

The co-occurrence of the most frequent landscape was shown in Figure 2 by using exploratory factor analysis. In particular, we have the following major landscapes: (1) AI techniques in diabetes diagnosis (machine learning, Support Vector Machine (SVM), Fuzzy) (red); (2) diabetes prediction using model (green); (3) risk factors prediction (yellow and orange); and (4) diabetes treatments.

In Table 5, we showcased the research topics that were constructed via the use of latent Dirichlet allocation where we scrutinized the most frequent words and titles for each topic and manually annotated the labels of the topics. The topics found to have the highest volumes of publications included: (1) AI application in diabetes prediction and diagnosis; (2) complications of diabetes prediction; (3) biomedicine and molecular biology in diabetes; (4) e-health for diabetes care, and (5) robot-assisted surgery for patients with diabetes.

**Figure 3:**
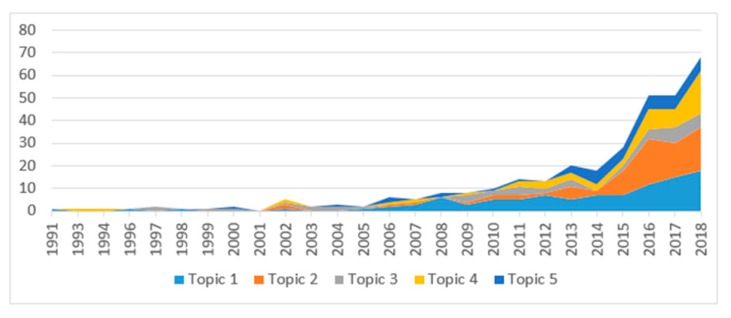
Changes in applications of Artificial Intelligence to diabetes research during 1991–2018.

In Figure 3, we illustrate the changes in research productivity over time. It shows that the number of publications related to AI in diabetes increased during the research period, especially from the beginning of the 21st century, especially in Topic 1 and Topic 2.

**Figure 4:**
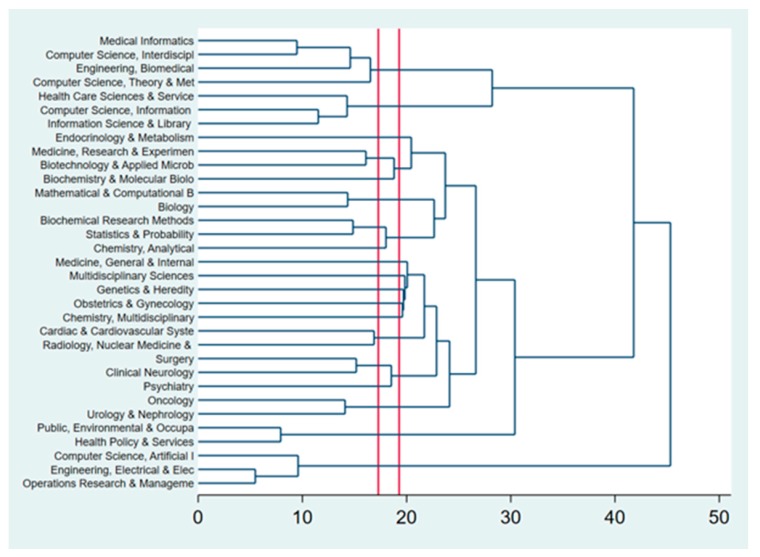
The clustering of research disciplines (WOS classification) used in Artificial Intelligence and Diabetes.

Based on WOS categories, we identified the dendrogram for those (Figure 4). The horizontal axis shows the dissimilarity between research areas. The vertical position of the split, shown by a short bar indicates the dissimilarity between research areas. AI in diabetes focused on the following research areas: (1) computer Application in health care and biomedicine for diabetes; (2) biotechnological investigation and physiological mechanisms of diabetes; (3) AI application in biomedicine and comorbidities of diabetes; (4) health policy and diabetes, and (5) technology and diabetes.

## 4. Discussion

The study clearly demonstrates a strong and increasing interest in the field of AI in the diagnosis and management of diabetes. This is evident by the increasing number of studies and a considerably higher extent of articles being published and applied. The gain in traction of such studies started from 2010 and has been steadily increasing to 2018, where a record number of 74 papers were published (Table 2). The nucleus driving the research and development of AI in diabetes is centered around developed countries mainly consisting of the United States, which contributed 44.1% of the publications with Ireland and Italy following behind (Table 3).

The study has found the top five emerging research domains (Table 5) pertaining to the application of AI in the diagnosis and management of diabetes: (1) uses of AI in the diagnosis of diabetes, (2) risk Assessment diabetes and its complications, (3) role of AI in novel treatments and monitoring in diabetes, (4) applications of telehealth and wearable technology in the daily management of diabetes, (5) robotic surgical outcomes with diabetes as a comorbid.

### 4.1. Uses of AI in the Diagnosis of Diabetes

Under this area of interest, the papers published in this category center around the diagnosis of diabetes using branches of AI. The intention of such research is not to replace the diagnosis work done by the doctors in healthcare, but to complement their efforts as such to optimize the patient’s diagnosis timings as well as to cut down on the workload burden.

There are many methods published under this category, examples include the diagnosis of type II diabetes via the analysis of parameters such as heart rate variability and arterial blood glucose alterations. They are performed using non-linear methods such as detrended fluctuation analysis (DFA) and Poincare plot to produce two metrics termed standard deviation ratio (SDR) and alpha-ratio. These two metrics are then fed into a machine-learning algorithm to dichotomize the subjects as diabetic or non-diabetic. The paper reports an accuracy of 94.7% in the correct categorization of subjects, which offers the possibility that it could be further developed as a non-invasive screening tool for predicting whether an individual has type II diabetes [15].

Using AI to diagnose different conditions such as diabetes is interesting, but it comes with drawbacks as well. The main problem with AI is that firstly, it needs to be well-trained, which would require a large number of patients in the training set. This is a problem because personal details protection and privacy is a concern. Another problem with AI diagnostics is that it needs to be consistent, replicable, and reliable, but so far many of the studies have not been applying the evidence-based approaches that are seen in established fields [16].

There have been questions about whether the use of AI can improve diagnostic ability in terms of efficacy and time reduction. For instance, a study found the AI model can detect breast cancer in whole slide images better than 11 pathologists, with the pathologists being limited by the allowed assessment time of one minute per image, while the AI is not limited by any factors. However, when the pathologists were given unlimited time, they performed similarly to the AI and detected more difficult cases more frequently than the computers [17]. This raised the possibility that AI may better be employed in the diagnosis of clear-cut simple cases, whereas more complex cases that require detailed assessment may be more worthwhile to be tackled by humans.

### 4.2. Risk Assessment of Diabetes and its Complications

A crucial principle in the management of type II diabetes is to retard the progression of the chronic disease as well as to prevent the onset of complications resulting from the condition. Many barriers to the optimal detection of the progression include patients defaulting appointments, non-compliance to medical advice, and financial barriers that include treatment costs and costs of a healthy diet [18]. Therefore, the development of AI in such a setting is beneficial to both doctors and patients as doctors will be able to foretell the course of the disease to individualize and cater to the proper management of the patient based on their risk.

A novel method of achieving the above objective is noted in a paper that studies the application of an artificial neural network model that aims to diagnose type II diabetes mellitus and establishes the relative importance of risk factors. The study was conducted on a cohort of 234 people who were diagnosed with type II diabetes mellitus using glycated hemoglobin levels. A multilayer perceptron artificial neural network that was utilized to highlight the demographic risk factors revealed that risk factors such as age, hypertension, waist circumference, body mass index, sedentary lifestyle, etc. were predictors of type II diabetes. However, the final analysis showed that the most important predictors and risk assessment of diabetes type II were waist circumference, age, BMI, hypertension, stress, smoking, and positive family history of type II diabetes [19].

These risk assessment models developed with the assistance of AI help to improve the detection/screening of diabetes type II by enabling the optimization of health resources for people who are known to be high risk, compared to screening the entire population at large. This not only improves the cost at a population level, but also the landscape of screening for chronic diseases such as diabetes.

### 4.3. Role of AI in Novel Treatments and Monitoring of Diabetes

Monitoring blood glucose levels is a crucial tool to prevent complications such as hypoglycemia, which may lead to coma, seizures, or even death. AI has been applied to analyze breath samples taken from different subjects to determine hypoglycemic states. This was inspired by diabetes alert dogs which could detect hypoglycemia based on their owner’s breath [20]. This presents a new way of monitoring glucose control and may help in the future to increase the uptake of self-glucose monitoring, that is currently mostly done with a glucometer, which is still quite troublesome for the patient and involves the pricking of the fingers, which some might not be comfortable with.

### 4.4. Applications of Telehealth and Wearable Technology in the Daily Management of Diabetes

In today’s day and age, telehealth and wearable technology is catching up and may soon be one of the revolutionary tools used in clinical healthcare. The benefits of such technology are not only convenience, but it also allows the patients to take up more responsibility for their own healthcare. Some of the applications include monitoring of the patient’s vitals through wearable technology such as a watch. In the future, it is possible for AI to be introduced to analyze biological information such as ultrasound scans or electrocardiography (ECG) obtained via the patient’s phone, and provide real-time analysis for the patient. These results can also be shared with the primary doctor or a hospital for further evaluation and action.

A mobile application for managing diabetic patients’ nutrition is currently being developed with the help of AI. This application combines AI techniques with a knowledge base constructed from the guidelines of the American Diabetes Association. This application recommends snacks based on the patient’s favorites as well as current diabetic condition, which may help to improve glycemic control and prevent episodes of hypoglycemia [21].

Promoting beneficial behavior change in people with or at risk of diabetes is also a promising area of application for AI [22]. Studies have shown that the use of fitness tracking applications in smartphones and/or wearable health devices such as smart watch have been found to be associated with increased physical activities and fitness level in diabetic patients [22,23]. The incorporation of behavior change strategies, for instance environmental restructuring, attitude adjusting, or identifying barriers to changes that are specific to each individual, which, argued Sullivan and Lachman to be possibly the most change influencing factors, but have had limited appearance in fitness and health applications [24], yet application in fitness/health applications would supposedly be easy and effective, given the power of AI to record and process a large amount information.

### 4.5. Robotic Surgery with Diabetes as a Co-Morbid

AI has also been able to assist surgeons in the form of robotic surgery known to come with greater precision, lesser complication rates, as well as quicker healing times. However, that is not all that AI can do in the field of surgery. Machine learning techniques have been known to be applied to the field of surgery, where it concluded that new-onset diabetes and preexisting diabetes are correlated with a decrease in long term survival after liver transplants. Using AI, this has enabled the comparison of different surgical techniques, their complication rates as well as to identify different factors that may affect the outcomes during and after the surgery [25].

### 4.6. Challenges in the Use of AI

The increasingly deeper integration of AI applications in the diagnosis, management, and prediction of diabetes has also come with challenges. Ethical issues, in particular the privacy and confidentiality of patient data, have been a topic of debate in existing literature [26]. It is noted that most of the AI technology is still under development and not yet utilized in clinical practice. For this to happen, AI needs to be more developed so that it can be as sensitive and specific as possible. This requires large datasets to train the AI or computer models. These datasets may consist of sensitive and confidential patient biodata such as their age, body mass index, etc. The use of wearable technology that constantly records data on behavior of diabetic users while also encouraging users to input their sensitive health data for it to provide more accurate, tailor-made suggestions would inherently expose users to the risk of having personal data leaked or being used for other purposes without their consent. Furthermore, with large datasets, massive amounts of data may lead to difficulty in human efforts to design intricate and perfectly logical models for specific clinical tasks or to provide appropriate, effective treatment, or behavior suggestions for users. Other common limitations of mathematical models such as the lack of model validations, especially for model of novel approach, data/measurement bias, issues in sample representations, and the occurrence of outliers, would also potentially be found with AI models. Therefore, further research and development would be needed to tackle these problems before AI may be reliably used in clinical practice as well as in diabetic care.

This bibliometric analysis has put forward several key developments in the field of AI in diabetes and has included large quantities of literature on the topic of interest. However, since the study has only included papers written in the English language, a bias towards Western countries may be present. In addition, another limitation of the study was that of a restriction to purely peer-reviewed research publications, which possibly could have affected the extensiveness of the analyzed results. Future studies may also benefit from applying sensitivity analysis for type I and type II diabetes when conducting research using the method applied in this study.

## 5. Conclusions

The application of AI in diabetes has evidently become more common in recent years, with AI-related technologies being found to assist from diagnosis and clinical treatment to daily management of diabetes. As a condition with severity depending heavily on the lifestyle and behavior of patients and those at risk, continuous monitoring and initiating specific treatments based on data of individual’s conditions and behavior as well as their specific surrounding would likely be effective, and is an area that AI applications can be seen as a promising solution. With such opportunities to enhance diabetes management also come challenges associated with the use of AI, of which privacy and confidentiality remain the major ones. These issues must be tackled and resolved before clinical use of AI-related technology is approved and available for the patient’s benefit.

## Figures and Tables

**Table 1:** Overview of analytical techniques utilized for each data type. WOS, Web of Science.

Type of Data	Unit of Analysis	Analytical Methods	Presentations of Results
Keywords, Countries	Words	Frequency of co-occurrence	Map of keywords clusters
Abstracts	Words	Exploratory factors analyses	Top 50 constructed research domains Clustering map of the landscapes constructed by these domains.
Abstracts	Papers	Latent Dirichlet Allocation	10 classifications of research topics
WOS ^1^ classification of research areas	WOS research areas	Frequency of co-occurrence	Dendrogram of research disciplines

^1^ WOS: Web of Science.

**Table 2:** General characteristics of publications.

Year Published	Total Number of Papers	Total Citations	Mean Cite Rate per Year	Total Usage Last 6 Months ^1^	Total Usage Last 5 Years ^1^	Mean Use Rate Last 6 Months ^2^	Mean Use Rate Last 5 Years ^2^
2018	74	60	0.8	405	739	5.5	2.0
2017	56	243	2.2	157	788	2.8	2.8
2016	57	400	2.3	61	656	1.1	2.3
2015	33	288	2.2	39	462	1.2	2.8
2014	22	196	1.8	29	403	1.3	3.7
2013	27	380	2.3	28	400	1.0	3.0
2012	17	135	1.1	9	117	0.5	1.4
2011	14	300	2.7	8	206	0.6	2.9
2010	12	343	3.2	8	107	0.7	1.8
2009	8	435	5.4	7	197	0.9	4.9
2008	8	291	3.3	8	75	1.0	1.9
2007	8	323	3.4	4	98	0.5	2.5
2006	8	213	2.0	9	130	1.1	3.3
2005	2	30	1.1	0	4	0.0	0.4
2004	5	321	4.3	3	56	0.6	2.2
2003	2	134	4.2	1	16	0.5	1.6
2002	5	177	2.1	0	21	0.0	0.8
2001	1	23	1.3	0	0	0.0	0.0
2000	4	44	0.6	0	10	0.0	0.5
1999	1	18	0.9	0	2	0.0	0.4
1998	2	48	1.1	0	5	0.0	0.5
1997	2	14	0.3	0	3	0.0	0.3
1996	1	22	1.0	0	4	0.0	0.8
1994	1	8	0.3	0	0	0.0	0.0
1993	1	2	0.1	0	0	0.0	0.0
1991	1	2	0.1	0	2	0.0	0.4

^1^ Total usage: Total number of download; ^2^ Use rate: Total number of downloads/Total number of papers.

**Table 3:** Number of papers by countries as study settings.

No.	Country Settings	Frequency	%	No.	Country	Frequency	%
1	United States	108	44.1%	19	Czech	2	0.8%
2	Ireland	25	10.2%	20	France	2	0.8%
3	Italy	15	6.1%	21	Netherlands	2	0.8%
4	India	14	5.7%	22	Singapore	2	0.8%
5	Australia	9	3.7%	23	United Arab Emirates	2	0.8%
6	Japan	8	3.3%	24	Antarctica	1	0.4%
7	Taiwan	6	2.4%	25	Brazil	1	0.4%
8	Spain	5	2.0%	26	Bulgaria	1	0.4%
9	United Kingdom	5	2.0%	27	Egypt	1	0.4%
10	Germany	4	1.6%	28	Greece	1	0.4%
11	Israel	4	1.6%	29	Jordan	1	0.4%
12	Switzerland	4	1.6%	30	Malaysia	1	0.4%
13	Iran	3	1.2%	31	Mexico	1	0.4%
14	Poland	3	1.2%	32	New Zealand	1	0.4%
15	Saudi Arabia	3	1.2%	33	Pakistan	1	0.4%
16	Austria	2	0.8%	34	Sweden	1	0.4%
17	Canada	2	0.8%	35	Tunisia	1	0.4%
18	China	2	0.8%	36	Turkey	1	0.4%

**Table 4:** Top 20 research domains emerged from exploratory factor analysis of all abstracts’ contents.

**No.**	**Name**	**Keywords**	**Eigenvalue**	**Frequency**	**% Cases**
1	Predict; Predictors	Prediction; Predictors; Predict; Random; Models; Learning; Machine; Records	2.89	173	74.39%
2	Events; Lead	Events; Lead; Developing; Detection; Potential; Treatment; Drug; Optimal; Medical; Work	2.2	114	71.95%
3	UCI ^1^; Fuzzy	UCI; Fuzzy; Heart; Disease; Proposed; Obtained; Problems	2.36	117	69.51%
4	Early; Rate	Early; Rate; Complications; Medical; Detection; Work	1.88	89	65.85%
5	Technique; Cross	Technique; Cross; Applied; Validation; Machine; Metabolic; Learning	2	132	64.63%
6	Support Vector Machine (SVM)	Vector; Support; SVM; Machine	3.04	101	59.76%
7	Development; Present	Development; Present; Show; Conditions; Mellitus; Real	2.33	75	58.54%
8	Classification	Classification; Predictive; Achieved	2.1	57	54.88%
9	Monitoring; Blood Glucose	Monitoring; Glucose; Short; Insulin; Blood; Long; Treatment	3.29	96	54.88%
10	Artificial Neural Network	Neural; Artificial; Network; Ann; Values; Parameters; DM ^2^; Obtained	3.58	121	53.66%
11	Large; Physicians	Large; Physicians; Screening; Processing; Performance; Long; Set; AUC	2.58	84	50.00%
12	Cost; Healthcare	Cost; Healthcare; Records; Predicting; Common; Risk	2.68	71	48.78%
13	Body Mass; Index	Mass; Body; Index; Testing; Surgery; Rate; Complications; Robotic	2.62	86	45.12%
14	Information; Develop	Information; Develop; Heart; Features; Long	2.47	60	43.90%
15	Clinical Decision	Decision; Tree; Clinical; Major	1.95	58	42.68%
16	Test; Neuropathy	Test; Neuropathy; Parameters; Component; Classifier; Accurate	2.23	59	41.46%
17	Feature Selection; Features	Feature; Selection; Features; Proposed; Paper	2.95	68	41.46%
18	Cohort; Hypertension	Cohort; Hypertension; Outcomes; Stage; Robotic; Surgery; Similar; Database; Complications	15.05	73	41.46%
19	Area; Curve (AUC) ^3^	Area; Curve; AUC; Identifying; Set; Evaluated	3.82	79	39.02%
20	Sensitivity, Specificity	Specificity; Sensitivity; Develop	1.85	42	26.83%

**^1^** UCI: Machine Learning Repository; **^2^** DM: Diabetes mellitus; **^3^** AUC: Area Under the Curve.

**Table 5:** 10 research topics classified by Latent Dirichlet Allocation

Year	Research areas	Frequency	%
Topic 1	AI application in diabetes prediction and diagnosis	100	31.1%
Topic 2	Complications of diabetes prediction	83	25.8%
Topic 3	Biomedicine and molecular biology in diabetes	43	13.4%
Topic 4	E-health for diabetes care	56	17.4%
Topic 5	Robot-assisted surgery for patients with diabetes	40	12.4%
